# Brain-Gut Interactions in IBS

**DOI:** 10.3389/fphar.2012.00127

**Published:** 2012-07-05

**Authors:** Jakub Fichna, Martin A. Storr

**Affiliations:** ^1^Department of Biomolecular Chemistry, Faculty of Medicine, Medical University of LodzLodz, Poland; ^2^Division of Gastroenterology, Department of Medicine, Ludwig Maximilians University of MunichMunich, Germany

**Keywords:** irritable bowel syndrome, brain-gut axis, pathophysiology, autonomic nervous system, hypothalamo-pituitary-adrenal axis

## Abstract

Irritable bowel syndrome (IBS) is a common gastrointestinal disorder with an estimated prevalence of 10–20%. Current understanding of the pathophysiology of IBS is incomplete due to the lack of a clearly identified pathological abnormality and due to the lack of reliable biomarkers. Possible mechanisms believed to contribute to IBS development and IBS like symptoms include physical stressors, such as infection or inflammation, psychological, and environmental factors, like anxiety, depression, and significant negative life events. Some of these mechanisms may involve the brain-gut axis (BGA). In this article we review the current knowledge on the possible involvement of the BGA in IBS and discuss new directions for potential future therapies of IBS.

## Introduction

Irritable bowel syndrome (IBS) is a common gastrointestinal (GI) disorder with an estimated prevalence of 10–20% (Philpott et al., 2011). According to Thompson et al. (2000) it accounts for about 3% of all general practice and up to 40% of all GI referrals. IBS causes considerable morbidity amongst its sufferers, who manifest with abdominal pain and altered stool consistency and frequency (Drossman and Dumitrascu, 2006; Lee et al., 2007; Adeyemo et al., 2010). Although not life-threatening, it is a heavy economic burden due to increased work absenteeism and impaired quality of life of its sufferers, as well as increased use of health care services (Sandler et al., 2002).

Current understanding of the pathogenesis of IBS is unsatisfactory due to the lack of demonstrable pathological abnormalities and reliable biomarkers. Traditionally, IBS has been considered a purely functional disorder. A hypothesis based on specimens obtained at endoscopy and in serological cytokine studies views IBS as a localized low grade inflammatory disorder with mast cells (MC) playing a particularly important role (Mayer and Collins, 2002; Philpott et al., 2011). An alternative hypothesis states that food allergy may be responsible (Atkinson et al., 2004). Most recently, the relationship between the neural and immunological networks within the gut and the bi-directional communication between the gut and the central nervous system (CNS), often related to as the brain-gut axis (BGA) attract most attention (Collins and Bercik, 2009).

In this review we focus on the disturbances in the BGA as a plausible cause of IBS. We overview the pathophysiological mechanisms contributing to symptom perception and generation and the endogenous systems involved. Particular attention is given to stress, emotion and psychological factors in the IBS pathogenesis. We also discuss new directions for potential future therapies of IBS based on discussed mechanisms.

## The Brain-Gut Axis

The BGA constitutes the enteric nervous system (ENS) and the gut wall in the periphery, the CNS, and the hypothalamo-pituitary-adrenal (HPA) axis (Collins and Bercik, 2009). The bi-directional communication between the gut and the CNS is based on the neural, endocrine and neuroimmune pathways. Neuronal pathways include afferent fibers originating in the dorsal root of the ganglia of the thoracic spinal cord (T1–T10) projecting to integrative cortical areas, such as the cerebral, anterior and posterior cingulate, insular, and amygdala cortices and efferent fibers to smooth muscle and glands, originating in nuclei within the brainstem, as well as S2–S4 spinal levels (parasympathetic) and in the lateral horn of the thoraco-lumbar spinal cord (T1–L3; sympathetic; Mulak and Bonaz, 2004; Gaman and Kuo, 2008; O’Mahony et al., 2011). The main pain signaling pathways in the BGA are the spinothalamic tracts and dorsal columns with descending supraspinal afferents originating from the rostral ventral medulla (Gaman and Kuo, 2008).

In physiological conditions, signals from the GI tract influence the brain, which in turn can exert changes in motility, secretion, and immune function (Mayer et al., 2006). The axis is therefore an important communication system for healthy regulation of food intake, digestion, gut sensations, and control of the bowel movements. Structural and functional disruptions in the BGA cause changes in perceptual and reflexive responses of the nervous system and may lead to GI disorders, including IBS, which often comorbid with chronic psychiatric diseases (Clarke et al., 2009; Gros et al., 2009).

### Structural and functional abnormalities in the central nervous system

Visceral hypersensitivity is a key mechanism underlying abdominal pain, one of the main symptoms of IBS (Azpiroz et al., 2007; Barbara et al., 2011). Visceral hypersensitivity is thought to be determined by central and peripheral mechanisms, as it may result from altered transmission within the gut wall, the spinal cord, or the brain. However, the specific contribution of the BGA components to hypersensitive responses in IBS remains unclear.

Direct imaging techniques were recently employed to detect the abnormalities in the structure and functioning of the brain and their possible implications in the pathology of IBS. There is only one structural magnetic resonance imaging (MRI) study (Davis et al., 2008), in which the thinning in the anterior midcingulate and insular cortex, structures important for perception of internal body states were observed in the IBS patients. These results were later confirmed by functional MRI (Blankstein et al., 2010). Although the underlying cause of cortical thinning was not elucidated, factors like decreased cell size, apoptosis of neural cells, death of glia and astrocytes, fewer dendritic spines, reduced synaptic density, and excitotoxicity related to enhanced glutamate signaling were suggested as possible contributors. Seminowicz et al. (2010) reported morphometric brain differences between female IBS patients and controls in terms of regional increases and decreases in gray matter density. These alterations occurred primarily in brain areas involved in attention and emotion modulation, as well as cortico-limbic pontine pain modulatory systems and in networks processing interoceptive information. Further studies of Blankstein et al. (2010) evidenced increased gray matter density in the hypothalamus of the IBS patients. Currently it is not possible to discern whether these changes are a predisposing factor for IBS or a secondary change after sustained visceral signals (Fukudo and Kanazawa, 2011).

In their excellent paper on imaging techniques used in the studies of brain-gut interactions, Rapps et al. (2008) reviewed the possible central mechanisms implicated in IBS and found published reports somewhat contradictory. The region that attracted most attention was the anterior cingulate cortex (ACC), one of the six most commonly reported cortical areas that display pain-evoked activity during acute stimulation in humans (Chen et al., 2011). ACC showed altered activity during rectal stimulation in IBS patients in comparison to healthy controls (Rapps et al., 2008 and citations therein). Interestingly, although greater pain by rectal balloon distension was reported by the IBS patients with a history of sexual or physical abuse, changes in their ACC activity were less pronounced than in other IBS patients and the controls (Ringel, 2002). In line with these observation was the study of Mertz et al. (2000), who demonstrated differential activation of the brain between IBS patients and controls. The ACC, the insula, the prefrontal cortex, and the thalamus were more activated in the IBS patients as compared with healthy controls and the pattern was related to the experience of individuals.

Hall et al. (2010) revealed differences in the central responses in health and in IBS to a single ramp-tonic distension of the colon across a distributed network of regions, involving sensory, striatal, limbic, and frontal areas. The IBS participants showed heightened activation of the ACC, suggesting increased affective responses to painful visceral stimuli. However, it was also observed that the activation of the thalamic, striatal, and dorsolateral prefrontal cortex (DLPFC) regions was relatively greater in control subjects, as compared to IBS patients, which may reflect increased ascending input to the brain, in particular to the cortex and a heightened arousal reaction to distension. Greater recruitment of the DLPFC by controls than IBS patients is consistent with the notion of abnormal descending modulation in IBS.

To further explore the central mechanisms of visceral hypersensitivity in IBS, Lawal et al. (2006) examined total cortical recruitment in response to subliminal (sub-conscious) stepped changes in distension pressure and observed that visceral hypersensitivity in IBS patients is due to increased afferent signaling to the brain, rather than altered processing at the level of the brain. However, the results of the study were later questioned, among others by Lackner et al. (2006), who showed that cognitive behavioral therapy in IBS patients is associated with a reduction of baseline activity in the ACC and accompanied by improvements of GI symptoms. Dorn et al. (2007) showed a contributory part of neurosensitivity in the form of enhanced activity with central neural networks independent of cognitive function.

The most novel findings of Chen et al. (2011) showing that the patients with IBS have white matter abnormalities in the insula, ACC, and other brain areas associated with pain, interoception, and homeostasis indicate that functional gray matter abnormalities in IBS patients are accompanied by white matter aberrations. The white matter deficiencies of the descending modulation of pain and dysfunction of the medial pain system may be responsible for the emotional aspect of pain in IBS.

In conclusion, as evidenced by the results of the meta-analysis performed by Tillisch et al. (2011), a greater engagement of regions associated with emotional arousal and endogenous pain modulation, but similar activation of regions involved in processing of visceral afferent information was observed in patients with IBS compared to controls. These results support a role for structural and functional abnormalities in the CNS in IBS.

### Cognitive–behavioral model of IBS

IBS is often considered a bio-psychosocial disorder (Engel, 1977; Camilleri and Choi, 1997; Drossman, 1998), which suggests that psychological (e.g., emotions, cognitions, and behavior), social (e.g., modeling, support), and physiological (e.g., cramps, bloating) factors may induce and exacerbate its symptoms (Toner et al., 1998; Mach, 2004). Individual cognitive and emotional responses to recurrent GI symptoms and associated life events may also affect the therapeutical efficacy of anti-IBS treatments (Kennedy et al., 2012).

### Central mechanisms

Abnormal activity within higher-order brain systems may alter cognitive and affective processes and contribute to both abnormal pain regulation and higher levels of anxiety and depression, typically reported in chronic pain conditions (Ribeiro et al., 2005) and IBS (Piche et al., 2011, and citations therein). The cognitive-behavioral model of IBS is focused particularly on emotional arousal and organism response to stress and the integrated network of structures, which include the hypothalamus, amygdala, and periaqueductal gray (PAG), as well as a number of neuromodulators and hormones.

Greenwood-Van et al. (2001) showed in animal models that there is a link between the central pathways mediating stress and anxiety and the mechanisms regulating the GI sensitivity. A key component of this link is the amygdala, which is known for its role in the regulation of emotional behavior and the expression of fear and anxiety. Further studies in rodents demonstrated that colonic sensitivity and motility are increased following fear conditioning (Gue et al., 1991; Tyler et al., 2007). In accordance, studies on IBS patients showed substantial activation of the hypothalamus and amygdala, as well as decreased activity of the antinociceptive PAG (Naliboff et al., 2001). More recent investigations employing rectosigmoid balloon distension in IBS patients have shown increased activity in the amygdala, insula, cingulate, and prefrontal cortex, which form a network of brain structures involved in regulating affective and sensory processes (Naliboff et al., 2003; Wilder-Smith et al., 2004; Myers and Greenwood-Van, 2009).

### Role of ANS and HPA axis

The autonomic nervous system (ANS) and the hypothalamus-pituitary-adrenal (HPA) axis are commonly regarded as the major components of the stress response system in the vertebrates. Alterations of this complex system have been linked to a variety of anxiety-related psychiatric disorders and stress-sensitive pain syndromes. Stress and stress-related psychosocial factors have also been proposed to act in IBS, particularly its post-infectious variety (PI-IBS), by overarching inflammation and the BGA (Arborelius et al., 1999; Gwee et al., 1999; Fukudo, 2007; Spiller and Garsed, 2009).

The correct function of the ANS and its cross-talk with CNS are important factors preventing from IBS. Disturbances at the ANS level, indicated by decreased parasympathetic and increased sympathetic activity and altered autonomic reflexes often occur in the IBS patients and account for the level of perception to GI stimuli and extra-intestinal symptoms (Azpiroz, 2002; Jarrett et al., 2003; Spaziani et al., 2008).

The key activator of the HPA axis is corticotrophin-releasing factor (CRF), an endogenous 41-amino acid neuropeptide secreted from endocrine cells in the paraventricular nucleus (PVN) of the hypothalamus (Aguilera et al., 2008). The action of CRF is mediated by the CRF1 and CRF2 receptors, which belong to the G protein-coupled receptor family (Kostich et al., 1998). CRF receptor activity can also be modulated by other peptides, like urocortins (UCN; Bale and Vale, 2004; Tache and Brunnhuber, 2008). In the mammalian brain three urocortins have been identified: UCN I, which binds to both receptors, and UCN II and UCN III, selectively binding to CRF2 receptor (Morin et al., 1999; Hsu and Hsueh, 2001; Lewis et al., 2001; Reyes et al., 2001; Bale and Vale, 2004; Dautzenberg et al., 2004). However, the neuroendocrine, autonomic, and behavioral responses to fear and stress are mediated exclusively by CRF and UCN I, which are selective CRF1 receptor ligands (Vale et al., 1981; Bale and Vale, 2004; Tache et al., 2009; Chen et al., 2011).

Corticotrophin-releasing factor and UCN I initiate the signaling cascade in the HPA axis by stimulating the anterior pituitary to secrete adrenocorticotropic hormone (ACTH), which in turn induces synthesis and secretion of glucocorticoids from the adrenal cortex. Growing evidence suggests that also the extra-hypothalamic CRF system is poised to play a critical role in both psychiatric and the BGA disorders (Lowry and Moore, 2006; Bravo et al., 2011).

In rodents, stress-induced release or exogenous administration of CRF and UCN I increased anxiety-like behaviors and stimulated colonic secretion, intestinal motility, and visceral sensitivity (Moreau et al., 1997; Slawecki et al., 1999; Saunders et al., 2002; Vetter et al., 2002; Million et al., 2003; Martinez et al., 2004; Tache et al., 2004, 2009). Johnson et al. (2010) provided evidence that elevated corticosterone levels affected the amygdala and significantly increased brain activation in response to colorectal distension (CRD) compared to that seen in cholesterol-treated controls. Elevated CRF expression was found in the thalamus of the rats exposed to neonatal maternal separation (Tjong et al., 2010). Deletion of the CRF1 gene using transgenic models or intraventricularly administered CRF1 antagonists had anxiolytic effects and attenuated stress- and CRF-induced alterations in gastric and colonic motor function (Smith et al., 1998; Million et al., 2003; Martinez and Tache, 2006; Trimble et al., 2007).

Only a limited number of studies in IBS patients measured basal and stimulated HPA axis hormone levels in response to meal, hormone challenge, or mental stress (Chang et al., 2009, and citations therein) and some of them demonstrated increased HPA axis responses in IBS compared to controls. Fukudo et al. (1998) observed that the intravenous injection of CRF in IBS patients induced exaggerated motility of the colon and increased visceral pain sensitivity compared with healthy controls, whereas administration of a non-selective CRF receptor antagonist ameliorated these responses (Lembo et al., 1996; Sagami et al., 2004). The recent study by Chang et al. (2009) showed that basal levels of plasma ACTH were significantly decreased, while both 24 h basal plasma cortisol levels and stress-induced cortisol levels were mildly elevated upon visceral stimulation in female IBS patients compared to controls, suggesting a dysregulation of the HPA axis in IBS. However, the role of the observed dysregulation of HPA axis in modulating IBS severity or abdominal pain remained unclear.

A meta-analysis performed by Tillisch et al. (2011) revealed that the central nucleus of amygdala indirectly activates the HPA axis and increases ACTH and glucocorticoid secretion via subcortical regions, which relay on PVN (Redgate and Fahringer, 1973; Feldman and Weidenfeld, 1998; Herman et al., 2003; Shepard et al., 2003). The CRF-dependent involvement of the amygdala in the induction of anxiety-like behavior, visceral hypersensitivity, altered bowel habits and other common feature of IBS has been later confirmed in animal studies (Tache et al., 2002; Myers and Greenwood-Van, 2007, 2010; Venkova et al., 2010). The hippocampus may also be involved in several aspects relevant to the IBS symptomatology, e.g., pain, anxiety, and stress (Prado and Roberts, 1985; Bannerman et al., 2004; Kwan et al., 2005; McEwen, 2007; Niddam et al., 2011). Saito et al. (2002) demonstrated that the induction of visceral pain by CRD increased the release of hippocampal noradrenaline in animal models. Niddam et al. (2011) observed abnormal hippocampal glutamatergic neurotransmission in IBS patients and inverse correlation between glutamate-glutamine concentrations and emotional stress indicators, which was not observed in healthy individuals. It remains possible that the observed hippocampal glutamatergic hypofunction could result from a generally impaired HPA axis tone or it could represent compensatory mechanisms of adaption to enhanced glucocorticoid feedback.

### Psychosocial factors and IBS

According to the cognitive-behavioral model, a history of abuse and other psychosocial factors may induce and aggravate symptoms of IBS, influence illness experience, and affect treatment outcome.

Ringel et al. (2008) showed that patients with IBS and a history of abuse had a significantly lower pain and urge thresholds and a greater tendency to report pain in response to aversive rectal distentions compared with patients with IBS or abuse history alone. However, neuro-sensory sensitivity remained unchanged. These observations suggest that the abuse history in IBS patients may affect central mechanisms of pain amplification or regional brain activation at sites linked to affect and attention, resulting in heightened awareness to visceral and somatic symptoms, greater pain reports, and greater clinical behavioral responses to painful visceral stimuli. Nevertheless, changes in peripheral signaling by nociceptive DRG neurons, including those innervating the colon cannot be excluded, as suggested by several animal studies (Khasar et al., 2008; Winston et al., 2010).

It was also observed that there is a higher prevalence of psychological and psychiatric disorders observed in IBS patients: depression, somatization disorder, generalized anxiety disorder, panic, and phobic disorders and coping difficulties (for review see, Arebi et al., 2008). Drossman et al. (1999) estimated that up to 70% of the patients referred to tertiary centers with IBS meet diagnostic criteria for anxiety or depression. However, Elsenbruch et al. (2006) revealed that women with IBS were characterized by an exaggerated anticipatory anxiety response at baseline, but essentially unaltered anxiety and neuroendocrine responses to a public speaking stressor. These results would suggest that IBS patients show essentially normal emotional responses when faced with challenging psychosocial situations.

Although well-evidenced, the impact of psychosocial factors on the neurochemical responsiveness of visceral nociceptive pathways and the physiological function of the GI remains unclear. It is possible that the psychosocial stressors and/or stressful early life events modulate the immune response of the gut to infectious agents and cause low level inflammation and mast cell infiltration and degranulation in the bowel (Barbara et al., 2004; Ohman and Simren, 2010; Chen et al., 2011; Philpott et al., 2011). This is supported by questionnaire-based studies indicating an increased prevalence of atopic diseases among IBS patients (Philpott et al., 2011, and citations therein) and a report published by Barbara et al. (2004), demonstrating that there is an increased number of degranulating MC in patients with IBS compared to that in the healthy controls. Increased mucosal immune activation and elevated blood concentrations in pro-inflammatory cytokines are also believed to impact the CNS functioning (for review see; Kennedy et al., 2012). Although these large molecules do not freely pass the blood-brain barrier, a number of studies have provided substantial evidence for their central mechanisms of action, sympathetic arousal and the HPA axis activation (Dinan et al., 2006).

In rodents, early life stress in the form of separation of neonates from the mother results in permanent changes in the CNS, which include unrestrained secretion of CRF and increased expression of its receptors (Owens and Nemeroff, 1993), increased regional norepinephrine release (Southwick et al., 1999), downregulation of β-receptors, decreased benzodiazepine receptor, and γ-aminobutryic acid type A receptor (Caldji et al., 2000). A significant increase in 5-HT-positive cell number and 5-HT content after CRD stimulation was also observed in the colon of animals, which experienced maternal separation (Ren et al., 2007). Videlock et al. (2009) demonstrated that IBS patients and controls with a history of early adverse life events (EAL) have a greater cortisol response to a visceral stressor compared to individuals without EAL, suggesting the involvement of the HPA axis.

## Current and Future Molecular Targets for IBS Treatment

Various classes of drugs, like 5-HT3 antagonists, tricyclic antidepressants (TCAs), selective serotonin reuptake inhibitors (SSRIs), gabapentinoids, CRF-1 antagonists, β3 adrenoceptor agonists, somatostatin, *N*-methyl d-aspartate receptor antagonists, or melatonin are currently in use for the treatment of visceral analgesia and other symptoms of IBS. However, new molecular targets for the future IBS therapeutics are also being investigated.

### Serotonin receptors

Serotonin (5-HT) is a key neurotransmitter and a signaling molecule that plays an important role in sensation, secretion, and absorption (for review see, Gershon and Tack, 2007; Garvin and Wiley, 2008). A number of studies reported altered serotonergic signaling activity in the brain and gut in IBS, including increase in plasma 5-HT in IBS-D (diarrhea-predominant) and PI-IBS, reduced levels in IBS-C (constipation-predominant) and changes in plasma and tissue levels of serotonin transporter protein (SERT; Dunlop et al., 2005; Atkinson et al., 2006; Zou et al., 2007; Camilleri, 2011). Drugs aimed at selective modulation of the 5-HT activity (SSRIs, 5-HT3, and 5-HT4 receptor antagonists) or both 5-HT and norepinephrine (NE) systems (serotonin-norepinephrine reuptake inhibitors, SNRIs, and tricyclic antidepressants, TCAs) have been used in the treatment of functional GI disorders, as well as in other chronic pain conditions, and psychiatric syndromes. New generation drugs with similar pharmacological profile may soon become novel efficient therapeutics in the treatment of IBS.

Several large clinical trials have demonstrated that serotonin receptor 5-HT3R antagonists, like alosetron, cilansetron, and ramosetron are among the most effective therapeutic options to date for both male and female IBS-D patients (Jarcho et al., 2008, and citations therein). The 5-HT3R antagonists alleviate specific IBS symptoms, such as frequent bowel movements, feelings of urgency, and chronic abdominal pain and discomfort, acting through central and peripheral mechanisms. Although the precise mechanisms underlying their effectiveness remain incompletely understood, symptom improvement associated with an interaction with dopamine, cholecystokinin, glutamate, acetylcholine, and GABA (for review see, Barnes et al., 2009) and a reduction in amygdala and emotional arousal circuit activity (Berman et al., 2002) have been suggested. Inhibition of the spinal cord c-fos expression by 5-HT3R antagonists in response to noxious CRD (Kozlowski et al., 2000) suggests that 5-HT3R plays a role in the transmission of noxious information within the spinal cord. Excess 5-HT released from enterochromaffin cells (EC) in the colonic mucosa of both unselected and PI-IBS patients (Spiller, 2007) and decreased expression of SERT (Coates et al., 2004) may also account to this phenomenon.

5-HT3 antagonist-based therapies require the implementation of a risk management plan, as ischemic colitis and complications of constipation may occur (Chang et al., 2010). Therefore, a novel class of compounds (of which the prototype is LX-1031) is being developed that directly inhibits 5-HT synthesis in EC cells, potentially reversing the underlying pathogenetic factor in conditions like IBS-D. Such compounds could become an alternative to the application of classical 5-HT3 receptor antagonists in the treatment of IBS.

Recently, partial 5-HT1 receptor agonists, like buspirone, and antagonists, like robalzotan tartrate monohydrate (AZD7371), attracted much attention as they displayed a potent analgesic effect in the CRD-induced visceral pain model in rats (Sivarao et al., 2004; Lindstrom et al., 2009). However, the clinical development of AZD7371 has been discontinued due to severe adverse events, including hallucinations and the inability to demonstrate significant efficacy in IBS patients compared with placebo (Drossman et al., 2008).

The 5-HT4 receptors in the GI tract are found on enteric neurons and smooth muscle cells. Stimulation of 5-HT4 receptors leads to acetylcholine release and prokinetic effects (Gershon and Tack, 2007). The early generation 5-HT4 receptor agonists, such as cisapride and tegaserod, reversed slow motility and relieved constipation, but they have been withdrawn because of cardiac or vascular adverse effects (Gershon and Tack, 2007). A number of novel 5-HT4 agonists have recently been obtained as potential treatments for patients with IBS-C and appear to be safer than earlier generation agents in these classes (Camilleri et al., 2008; Manini et al., 2010).

The 5-HT7 and 5-HT2B receptors are yet another potential serotonergic target for future IBS treatment. The 5-HT7 receptors are present in humans and other animals and are linked with depression, circadian rhythm, neuroendocrine function, affective behavior and body temperature regulation (for review see, Vanhoenacker et al., 2000). They play an important role in regulating smooth muscle relaxation in the GI and nociceptive pathways (Carter et al., 1995; Meuser et al., 2002) and may thus be involved in the pathological mechanisms of GI dyskinesia, abdominal pain, and visceral paresthesia in IBS. It was demonstrated that 5-HT7 receptors also mediate stress and glucocorticoid-induced effects on hippocampal neurogenesis, which have been implicated in mood. Meanwhile, 5-HT2B receptor blockade was shown to reduce significantly pain behaviors in response to CRD (O’Mahony et al., 2010a).

Recent studies demonstrated that serotonergic neurotransmission can be markedly affected by CRF acting in a CRF receptor-dependent manner (Cryan et al., 2005; Valentino and Commons, 2005). The injection of low doses of CRF in the dorsal raphe nucleus (DRN) reduced the discharge rate of serotonergic neurons in the striatum (Kirby et al., 2000) and the nucleus accumbens (Lukkes et al., 2008) and at a higher dose increased striatal 5-HT release (Price et al., 1998). Additionally, 5-HT levels in the hippocampus were increased by i.c.v. administration of low and high doses of CRF (Penalva et al., 2002). These data suggest a close correlation between the serotonergic system and CRF, which may be taken into consideration when novel anti-IBS therapies are designed.

### Benzodiazepine receptors

One of the newly targeted classes of drugs for the treatment of visceral pain are benzodiazepine (BZD) receptor modulators. BZD receptors are located in subcortical and hypothalamic regions and appear important in controlling autonomic function, such as motor and sensory activity of the gut (for review see, Salari and Abdollahi, 2011). In addition, activation of the central BZD receptors affects GABA interaction with central GABA-A receptors and may influence the ANS, dorsal vagal nuclei, and the ENS. Peripheral BZD receptors were identified on immune cells and other peripheral tissues and may be involved in cell proliferation and immunomodulation (for review see, Zisterer and Williams, 1997).

The BZD receptors and their ligands, which belong to an important regulatory network between the CNS, behavior, and immune response, may thus become an attractive target for future IBS treatments. Recently, a novel BZD receptor ligand dextofisopam was developed for the management of IBS-D (Grundmann et al., 2010) and is currently under investigation.

### Neurokinin receptors

Substance P (SP) and the neurokinin-1 receptors (NK1R) are located throughout the BGA, including peripheral, spinal, supraspinal, and cortical sites of visceral afferent pathways, as well as brain regions involved in emotional arousal and autonomic function (Tillisch et al., 2012, and citations therein). It was observed that SP and NK1R signaling play an important role in nociceptive responses (hyperalgesia) and the autonomic and behavioral responses to stress in animals and humans.

Recent study by Tillisch et al. (2012) revealed that a 3-week treatment with a novel NK1R antagonist reduced activation of key regions of both the interoceptive afferent and emotional arousal network in response to noxious and non-noxious visceral stimulus in female IBS patients, causing a large decrease in pain-induced negative affect and decreased anxiety and pain ratings. This positive correlation suggests a potential for use of NK1R antagonists in IBS patients to decrease pain related distress.

### Brain-derived neurotrophic factor

Neurotrophins promote neuronal survival along with the growth and differentiation of new neurons and synapses. Brain-derived neurotrophic factor (BDNF) may be involved in the integration of excitatory and inhibitory neurotransmission and emerging evidence suggests that amygdaloid BDNF can regulate anxiety-like behaviors (Slack et al., 2004; Pandey et al., 2006).

Yu et al. (2012) recently observed a significant upregulation of BDNF in the colonic mucosa and structural alterations of mucosal innervation in biopsies from patients with IBS, as compared with controls. The enhanced expression of BDNF was closely correlated with the degree of abdominal pain in IBS. These results suggest that endogenous BDNF released in response to inflammation contributes to the development of central sensitization and thus plays a pathophysiological role in the altered gut sensation in IBS. Furthermore, the upregulation of BDNF may also play a role in the structural alterations of mucosal nerve fibers in patients with IBS. Inhibition of the BDNF system could therefore be beneficial for the alleviation of symptoms in the IBS patients.

### Sex steroid receptors

Because of the sex differences in perceptual responses and a female predominance of the disorder, attention has been drawn to the role of sex steroids, in particular ovarian hormones, in the development of IBS. Previous reports revealed that women with IBS often report exacerbation of symptoms, including visceral and somatic sensitivity during menses (Kane et al., 1998; Mayer et al., 1999; Houghton et al., 2002; Chang et al., 2006; Gustafsson and Greenwood-Van, 2011) and show greater, compared to men, activation of brain areas associated with affective responses including the amygdala and cingulate cortex (Berman et al., 2000; Naliboff et al., 2003). In contrast, male IBS patients show less visceral hypersensitivity than female patients, but have greater sympathetic nervous system responses measured by skin conductance, and decreased cardiovagal activity measured by heart rate variability compared to female IBS patients (Tillisch et al., 2005) and male controls.

Although ovarian steroid receptor levels are higher in some regions of the female brain (Greco et al., 2001; Milner et al., 2008), progesterone and estradiol-induced visceral hypersensitivity does not appear to be sex specific, as males also showed increased visceral sensitivity following hormone implantation on the amygdala (Myers et al., 2011). However, the amygdala may still represent the key supraspinal site mediating the actions of ovarian hormones on visceral pain in both males and females and account for differences in symptom generation in male and female IBS patients (Naliboff et al., 2003; Labus et al., 2008; Kilpatrick et al., 2010). The amygdala may thus become an interesting target for the IBS treatment and alleviation of pain.

### Toll-like receptors

Toll-like receptors (TLRs) have been localized on mucosal surfaces, including the colonic epithelial cells, and their expression is increased in the colonic mucosa of rat models of visceral hypersensitivity and mucosal biopsies from IBS patients (McKernan et al., 2009; Brint et al., 2011). TLRs are activated by various bacterial and viral cell components (Takeuchi and Akira, 2010), which stimulate transcription of inflammatory cytokines, like IL-1b, IL-6, and TNFa and affect transmission in the spinal cord, resulting in central sensitization and hyperalgesia (for review seel, Akira and Takeda, 2004; Arebi et al., 2008). Cytokines are also known to cross the blood-brain barrier, to affect the HPA axis and stress response and to stimulate secretion of CRH in rat, as well as in humans (for review see, John and Buckingham, 2003; Dantzer et al., 2008).

Recently, McKernan et al. (2011) demonstrated that TLR agonist-induced cytokine and cortisol release was markedly enhanced in stimulated whole blood from IBS patients compared with healthy controls. These results point out at the TLR as possible targets in the treatment of IBS.

### Receptors for acetylcholine and catecholamines

There is an increasing evidence for the beneficiary role of cholinergic, dopaminergic, and noradrenergic pathways in regulating immunity and cytokine production in IBS, suggesting a positive influence of acetylcholine and catecholamines on the IBS symptoms (Dinan et al., 2008; Rosas-Ballina and Tracey, 2009). However, adrenaline was shown to act directly through adrenergic receptors on DRG neurons or indirectly by increasing levels of pronociceptive mediators following immune activation in the colon or repeated stress, thus increasing the excitability of the neurons and exacerbating pain sensation (Khasar et al., 2008; Winston et al., 2010; Ibeakanma et al., 2011). In contrast, no significant differences in NE responses to sigmoidoscopy were observed in women with IBS-D compared to healthy women (Chang et al., 2009).

These conflicting results point at the necessity of further studies on the involvement of cholinergic, dopaminergic, and adrenergic receptors and their ligands in development of IBS and their possible therapeutical application.

## Past, Present, and Future of Anti-IBS Drugs Targeting the Brain-Gut Axis

For most IBS patients with mild symptoms, lifestyle, and dietary changes may be sufficient; for more moderate symptoms, medications that act on the gut (e.g., anticholinergics, peripheral 5-HT agents) can be considered. However, patients who suffer from severe IBS, characterized by increased levels of pain, poorer quality of life, psychosocial difficulties, or co-morbidity with mood disturbances are usually refractory to first- and second-line therapies (Drossman et al., 2000; Grover and Drossman, 2011). The bi-directional communication between the brain and the gut opens up new treatment possibilities for these patients and directs us to novel pharmacological targets for the anti-IBS drugs.

Almost all IBS patients could benefit from centrally acting treatments, like therapies focused on teaching better stress coping strategies, both at a cognitive and behavioral level (for review see, Larauche et al., 2012), or psychotropic agents. Some of the TCAs, SSRIs, SNRIs, or BZDs have already been employed in the treatment of IBS and proved effective in symptom relief via mood stabilization, modulation of pain perception and amelioration of GI motility and secretion (Ford et al., 2009; Grover and Drossman (2011) estimate that at least every one in eight patients with IBS is offered an antidepressant). However, the effects of psychotropic agents on bowel symptoms and visceral hypersensitivity in IBS patients have been less robust and less consistent than the benefits reported for global symptoms and abdominal pain/discomfort (Chey et al., 2011). Furthermore, psychotropic agents are not free from undesired side effects. TCAs display anticholinergic properties, including constipation, tachycardia, urinary retention, and xerostomia; patients may also encounter central side effects including sedation, insomnia, agitation, and nightmares (Chey et al., 2011). Compared to TCAs, SSRIs have fewer side effects, but do not improve bloating or visceral pain (Tack et al., 2006). BZDs are used routinely in anxiety disorders, but their efficacy in symptom relief of IBS is under debate (Drossman et al., 2002). New generation of psychotropic agents is therefore anticipated.

Efficacious and safe serotonergic agents may also become future drugs in the treatment of IBS. Recently, novel mixed 5-HT1A agonists/5-HT3 antagonists, 5-HT1B/D agonists, and 5-HT2B antagonists have been proposed as new therapeutics for IBS (Tack et al., 2000; Mulak and Paradowski, 2006; Vera-Portocarrero et al., 2008; Asagarasu et al., 2009; O’Mahony et al., 2010b).

Other endogenous systems, which may become possible new targets in the IBS therapy, include GABA-B, CRF, NK, cannabinoid, and opioid receptors and their ligands. Preliminary data suggest that anxiolytic activity of GABA-ergic agent, gabapentin may be efficient in reducing central sensitization in hyperalgesia (for review see, Camilleri and Andresen, 2009). CRF receptor antagonists have also been proposed as a potential treatment of IBS (Martinez and Tache, 2006; Tache et al., 2009). However, due the failure of treatment with a CRFR1 antagonists to alter colonic transit and the global improvement scale in IBS patients (Sweetser et al., 2009), further studies are required.

The potential use of cannabinoid and opioid receptor ligands as anti-IBS agents has also been considered and has been reviewed in detail elsewhere (Fichna et al., 2009; Izzo and Sharkey, 2010).

## Conclusion

In summary, there is striking evidence of a crucial involvement of the BGA in the development of IBS and IBS like symptoms. Though the role of the BGA is not fully understood, some concepts are at an advanced stage and allow speculation on possible future treatment options. Future research needs to identify the exact involvement of the discussed neurotransmitter systems and to identify at which level pharmacological treatment may be beneficial to patients with IBS.

## Conflict of Interest Statement

The authors declare that the research was conducted in the absence of any commercial or financial relationships that could be construed as a potential conflict of interest.

## Abbreviations

ACC, anterior cingulate cortex; ACTH, adrenocorticotropic hormone; ANS, autonomic nervous system; BDNF, brain-derived neurotrophic factor; BZD, benzodiazepine; CNS, central nervous system; CRD, colorectal distension; CRF, corticotrophin-releasing factor; DLPFC, dorsolateral prefrontal cortex; DRG, dorsal root ganglia; DRN, dorsal raphe nucleus; ENS, enteric nervous system; GI, gastrointestinal; HPA axis, hypothalamo-pituitary-adrenal axis; IBS, irritable bowel syndrome; MC, mast cells; MRI, magnetic resonance imaging; NE, norepinephrine; PAG, periaqueductal gray; PVN, paraventricular nucleus; SERT, serotonin transporter protein; SNRI, serotonin-norepinephrine reuptake inhibitor; SSRI, selective serotonin re-uptake inhibitor; TCA, tricyclic antidepressant; UCN, urocortin.

## References

[B1] Adeyemo M. A. Spiegel B. M. Chang L. (2010). Meta-analysis: do irritable bowel syndrome symptoms vary between men and women? Aliment. Pharmacol. Ther. 32, 738–755 10.1111/j.1365-2036.2010.04409.x 20662786 PMC2932820

[B2] Aguilera G. Subburaju S. Young S. Chen J. (2008). The parvocellular vasopressinergic system and responsiveness of the hypothalamic pituitary adrenal axis during chronic stress. Prog. Brain Res. 170, 29–39 10.1016/S0079-6123(08)00403-2 18655869 PMC2536760

[B3] Akira S. Takeda K. (2004). Toll-like receptor signalling. Nat. Rev. Immunol. 4, 499–511 10.1038/nri1391 15229469

[B4] Arborelius L. Owens M. J. Plotsky P. M. Nemeroff C. B. (1999). The role of corticotropin-releasing factor in depression and anxiety disorders. J. Endocrinol. 160, 1–12 10.1677/joe.0.160R001 9854171

[B5] Arebi N. Gurmany S. Bullas D. Hobson A. Stagg A. Kamm M. (2008). Review article: the psychoneuroimmunology of irritable bowel syndrome – an exploration of interactions between psychological, neurological and immunological observations. Aliment. Pharmacol. Ther. 28, 830–840 10.1111/j.1365-2036.2008.03801.x 18637004

[B6] Asagarasu A. Matsui T. Hayashi H. Tamaoki S. Yamauchi Y. Sato M. (2009). Design and synthesis of piperazinylpyridine derivatives as novel 5-HT1A agonists/5-HT3 antagonists for the treatment of irritable bowel syndrome (IBS). Chem. Pharm. Bull. 57, 34–42 10.1248/cpb.57.34 19122313

[B7] Atkinson W. Lockhart S. Whorwell P. J. Keevil B. Houghton L. A. (2006). Altered 5-hydroxytryptamine signaling in patients with constipation- and diarrhea-predominant irritable bowel syndrome. Gastroenterology 130, 34–43 10.1053/j.gastro.2005.09.031 16401466

[B8] Atkinson W. Sheldon T. A. Shaath N. Whorwell P. J. (2004). Food elimination based on IgG antibodies in irritable bowel syndrome: a randomised controlled trial. Gut 53, 1459–1464 10.1136/gut.2003.037697 15361495 PMC1774223

[B9] Azpiroz F. (2002). Gastrointestinal perception: pathophysiological implications. Neurogastroenterol. Motil. 14, 229–239 10.1046/j.1365-2982.2002.00324.x 12061907

[B10] Azpiroz F. Bouin M. Camilleri M. Mayer E. A. Poitras P. Serra J. Spiller R. C. (2007). Mechanisms of hypersensitivity in IBS and functional disorders. Neurogastroenterol. Motil. 19(1 Suppl.), 62–88 10.1111/j.1365-2982.2006.00875.x 17280586

[B11] Bale T. L. Vale W. W. (2004). CRF and CRF receptors: role in stress responsivity and other behaviors. Annu. Rev. Pharmacol. Toxicol. 44, 525–557 10.1146/annurev.pharmtox.44.101802.121410 14744257

[B12] Bannerman D. M. Rawlins J. N. McHugh S. B. Deacon R. M. Yee B. K. Bast T. Zhang W. N. Pothuizen H. H. Feldon J. (2004). Regional dissociations within the hippocampus – memory and anxiety. Neurosci. Biobehav. Rev. 28, 273–283 10.1016/j.neubiorev.2004.03.004 15225971

[B13] Barbara G. Cremon C. De Giorgio R. Dothel G. Zecchi L. Bellacosa L. Carini G. Stanghellini V. Corinaldesi R. (2011). Mechanisms underlying visceral hypersensitivity in irritable bowel syndrome. Curr. Gastroenterol. Rep. 13, 308–315 10.1007/s11894-011-0195-7 21537962

[B14] Barbara G. Stanghellini V. De Giorgio R. Cremon C. Cottrell G. S. Santini D. Pasquinelli G. Morselli-Labate A. M. Grady E. F. Bunnett N. W. Collins S. M. Corinaldesi R. (2004). Activated mast cells in proximity to colonic nerves correlate with abdominal pain in irritable bowel syndrome. Gastroenterology 126, 693–702 10.1053/j.gastro.2003.11.055 14988823

[B15] Barnes N. M. Hales T. G. Lummis S. C. Peters J. A. (2009). The 5-HT3 receptor – the relationship between structure and function. Neuropharmacology 56, 273–284 10.1016/j.neuropharm.2008.08.003 18761359 PMC6485434

[B16] Berman S. Munakata J. Naliboff B. D. Chang L. Mandelkern M. Silverman D. Kovalik E. Mayer E. A. (2000). Gender differences in regional brain response to visceral pressure in IBS patients. Eur. J. Pain 4, 157–172 10.1053/eujp.2000.0167 10957697

[B17] Berman S. M. Chang L. Suyenobu B. Derbyshire S. W. Stains J. Fitzgerald L. Mandelkern M. Hamm L. Vogt B. Naliboff B. D. Mayer E. A. (2002). Condition-specific deactivation of brain regions by 5-HT3 receptor antagonist alosetron. Gastroenterology 123, 969–977 10.1053/gast.2002.35990 12360456

[B18] Blankstein U. Chen J. Diamant N. E. Davis K. D. (2010). Altered brain structure in irritable bowel syndrome: potential contributions of pre-existing and disease-driven factors. Gastroenterology 138, 1783–1789 10.1053/j.gastro.2009.12.043 20045701

[B19] Bravo J. A. Dinan T. G. Cryan J. F. (2011). Alterations in the central CRF system of two different rat models of comorbid depression and functional gastrointestinal disorders. Int. J. Neuropsychopharmacol. 14, 666–683 10.1017/S1461145710000994 20860876

[B20] Brint E. K. MacSharry J. Fanning A. Shanahan F. Quigley E. M. (2011). Differential expression of toll-like receptors in patients with irritable bowel syndrome. Am. J. Gastroenterol. 106, 329–336 10.1038/ajg.2010.438 21102570

[B21] Caldji C. Diorio J. Meaney M. J. (2000). Variations in maternal care in infancy regulate the development of stress reactivity. Biol. Psychiatry 48, 1164–1174 10.1016/S0006-3223(00)01084-2 11137058

[B22] Camilleri M. (2011). LX-1031, a tryptophan 5-hydroxylase inhibitor, and its potential in chronic diarrhea associated with increased serotonin. Neurogastroenterol. Motil. 23, 193–200 10.1111/j.1365-2982.2011.01749.x 21159063 PMC3076306

[B23] Camilleri M. Andresen V. (2009). Current and novel therapeutic options for irritable bowel syndrome management. Dig. Liver Dis. 41, 854–862 10.1016/j.dld.2009.07.009 19665953 PMC2783342

[B24] Camilleri M. Choi M. G. (1997). Review article: irritable bowel syndrome. Aliment. Pharmacol. Ther. 11, 3–15 10.1046/j.1365-2036.1997.84256000.x 9042970

[B25] Camilleri M. Kerstens R. Rykx A. Vandeplassche L. (2008). A placebo-controlled trial of prucalopride for severe chronic constipation. N. Engl. J. Med. 358, 2344–2354 10.1056/NEJMoa0800670 18509121

[B26] Carter D. Champney M. Hwang B. Eglen R. M. (1995). Characterization of a postjunctional 5-HT receptor mediating relaxation of guinea-pig isolated ileum. Eur. J. Pharmacol. 280, 243–250 10.1016/0014-2999(95)00195-Q 8566092

[B27] Chang L. Mayer E. A. Labus J. S. Schmulson M. Lee O. Y. Olivas T. I. Stains J. Naliboff B. D. (2006). Effect of sex on perception of rectosigmoid stimuli in irritable bowel syndrome. Am. J. Physiol. Regul. Integr. Comp. Physiol. 291, R277–R284 10.1152/ajpregu.00729.2005 16574882

[B28] Chang L. Sundaresh S. Elliott J. Anton P. A. Baldi P. Licudine A. Mayer M. Vuong T. Hirano M. Naliboff B. D. Ameen V. Z. Mayer E. A. (2009). Dysregulation of the hypothalamic-pituitary-adrenal (HPA) axis in irritable bowel syndrome. Neurogastroenterol. Motil. 21, 149–159 10.1111/j.1365-2982.2009.01328.x 18684212 PMC2745840

[B29] Chang L. Tong K. Ameen V. (2010). Ischemic colitis and complications of constipation associated with the use of alosetron under a risk management plan: clinical characteristics, outcomes, and incidences. Am. J. Gastroenterol. 105, 866–875 10.1038/ajg.2010.49 20197759

[B30] Chen J. Y. Blankstein U. Diamant N. E. Davis K. D. (2011). White matter abnormalities in irritable bowel syndrome and relation to individual factors. Brain Res. 1392, 121–131 10.1016/j.brainres.2011.03.068 21466788

[B31] Chey W. D. Maneerattaporn M. Saad R. (2011). Pharmacologic and complementary and alternative medicine therapies for irritable bowel syndrome. Gut Liver 5, 253–266 10.5009/gnl.2011.5.3.253 21927652 PMC3166664

[B32] Clarke G. Quigley E. M. Cryan J. F. Dinan T. G. (2009). Irritable bowel syndrome: towards biomarker identification. Trends Mol. Med. 15, 478–489 10.1016/j.molmed.2009.08.001 19811951

[B33] Coates M. D. Mahoney C. R. Linden D. R. Sampson J. E. Chen J. Blaszyk H. Crowell M. D. Sharkey K. A. Gershon M. D. Mawe G. M. Moses P. L. (2004). Molecular defects in mucosal serotonin content and decreased serotonin reuptake transporter in ulcerative colitis and irritable bowel syndrome. Gastroenterology 126, 1657–1664 10.1053/j.gastro.2004.03.013 15188158

[B34] Collins S. M. Bercik P. (2009). The relationship between intestinal microbiota and the central nervous system in normal gastrointestinal function and disease. Gastroenterology 136, 2003–2014 10.1053/j.gastro.2009.01.075 19457424

[B35] Cryan J. F. Valentino R. J. Lucki I. (2005). Assessing substrates underlying the behavioral effects of antidepressants using the modified rat forced swimming test. Neurosci. Biobehav. Rev. 29, 547–569 10.1016/j.neubiorev.2005.03.008 15893822

[B36] Dantzer R. O’Connor J. C. Freund G. G. Johnson R. W. Kelley K. W. (2008). From inflammation to sickness and depression: when the immune system subjugates the brain. Nat. Rev. Neurosci. 9, 46–56 10.1038/nrn2297 18073775 PMC2919277

[B37] Dautzenberg F. M. Higelin J. Wille S. Brauns O. (2004). Molecular cloning and functional expression of the mouse CRF2(a) receptor splice variant. Regul. Pept. 121, 89–97 10.1016/j.regpep.2004.04.009 15256278

[B38] Davis K. D. Pope G. Chen J. Kwan C. L. Crawley A. P. Diamant N. E. (2008). Cortical thinning in IBS: implications for homeostatic, attention, and pain processing. Neurology 70, 153–154 10.1212/01.wnl.0000295509.30630.10 17959767

[B39] Dinan T. G. Clarke G. Quigley E. M. Scott L. V. Shanahan F. Cryan J. Cooney J. Keeling P. W. (2008). Enhanced cholinergic-mediated increase in the pro-inflammatory cytokine IL-6 in irritable bowel syndrome: role of muscarinic receptors. Am. J. Gastroenterol. 103, 2570–2576 10.1111/j.1572-0241.2008.01871.x 18785949

[B40] Dinan T. G. Quigley E. M. Ahmed S. M. Scully P. O’Brien S. O’Mahony L. O’Mahony S. Shanahan F. Keeling P. W. (2006). Hypothalamic-pituitary-gut axis dysregulation in irritable bowel syndrome: plasma cytokines as a potential biomarker? Gastroenterology 130, 304–311 10.1053/j.gastro.2005.11.033 16472586

[B41] Dorn S. D. Palsson O. S. Thiwan S. I. Kanazawa M. Clark W. C. van Tilburg M. A. Drossman D. A. Scarlett Y. Levy R. L. Ringel Y. Crowell M. D. Olden K. W. Whitehead W. E. (2007). Increased colonic pain sensitivity in irritable bowel syndrome is the result of an increased tendency to report pain rather than increased neurosensory sensitivity. Gut 56, 1202–1209 10.1136/gut.2006.117390 17483191 PMC1954968

[B42] Drossman D. A. (1998). Presidential address: gastrointestinal illness and the biopsychosocial model. Psychosom. Med. 60, 258–267 9625212 10.1097/00006842-199805000-00007

[B43] Drossman D. A. Camilleri M. Mayer E. A. Whitehead W. E. (2002). AGA technical review on irritable bowel syndrome. Gastroenterology 123, 2108–2131 10.1053/gast.2002.37095 12454866

[B44] Drossman D. A. Creed F. H. Olden K. W. Svedlund J. Toner B. B. Whitehead W. E. (1999). Psychosocial aspects of the functional gastrointestinal disorders. Gut 45(Suppl. 2), II25–II30 10.1136/gut.45.2008.ii25 10457041 PMC1766694

[B45] Drossman D. A. Danilewitz M. Naesdal J. Hwang C. Adler J. Silberg D. G. (2008). Randomized, double-blind, placebo-controlled trial of the 5-HT1A receptor antagonist AZD7371 tartrate monohydrate (robalzotan tartrate monohydrate) in patients with irritable bowel syndrome. Am. J. Gastroenterol. 103, 2562–2569 10.1111/j.1572-0241.2008.02115.x 18775020

[B46] Drossman D. A. Dumitrascu D. L. (2006). Rome III new standard for functional gastrointestinal disorders. J. Gastrointestin. Liver Dis. 15, 237–241 17013448

[B47] Drossman D. A. Whitehead W. E. Toner B. B. Diamant N. Hu Y. J. Bangdiwala S. I. Jia H. (2000). What determines severity among patients with painful functional bowel disorders? Am. J. Gastroenterol. 95, 974–980 10.1111/j.1572-0241.2000.01936.x 10763947

[B48] Dunlop S. P. Coleman N. S. Blackshaw E. Perkins A. C. Singh G. Marsden C. A. Spiller R. C. (2005). Abnormalities of 5-hydroxytryptamine metabolism in irritable bowel syndrome. Clin. Gastroenterol. Hepatol. 3, 349–357 10.1016/S1542-3565(04)00726-8 15822040

[B49] Elsenbruch S. Lucas A. Holtmann G. Haag S. Gerken G. Riemenschneider N. Langhorst J. Kavelaars A. Heijnen C. J. Schedlowski M. (2006). Public speaking stress-induced neuroendocrine responses and circulating immune cell redistribution in irritable bowel syndrome. Am. J. Gastroenterol. 101, 2300–2307 10.1111/j.1572-0241.2006.00837.x 16952284

[B50] Engel G. L. (1977). The need for a new medical model: a challenge for biomedicine. Science 196, 129–136 10.1126/science.847460 847460

[B51] Feldman S. Weidenfeld J. (1998). The excitatory effects of the amygdala on hypothalamo-pituitary-adrenocortical responses are mediated by hypothalamic norepinephrine, serotonin, and CRF-41. Brain Res. Bull. 45, 389–393 10.1016/S0361-9230(97)00384-5 9527013

[B52] Fichna J. Schicho R. Janecka A. Zjawiony J. K. Storr M. (2009). Selective natural kappa opioid and cannabinoid receptor agonists with a potential role in the treatment of gastrointestinal dysfunction. Drug News Perspect. 22, 383–392 10.1358/dnp.2009.22.7.1400219 19890495

[B53] Ford A. C. Talley N. J. Schoenfeld P. S. Quigley E. M. Moayyedi P. (2009). Efficacy of antidepressants and psychological therapies in irritable bowel syndrome: systematic review and meta-analysis. Gut 58, 367–378 10.1136/gut.2008.163162 19001059

[B54] Fukudo S. (2007). Role of corticotropin-releasing hormone in irritable bowel syndrome and intestinal inflammation. J. Gastroenterol. 42(Suppl. 17), 48–51 10.1007/s00535-006-1942-7 17238026

[B55] Fukudo S. Kanazawa M. (2011). Gene, environment, and brain-gut interactions in irritable bowel syndrome. J. Gastroenterol. Hepatol. 26(Suppl. 3), 110–115 10.1111/j.1440-1746.2011.06631.x 21443722

[B56] Fukudo S. Nomura T. Hongo M. (1998). Impact of corticotropin-releasing hormone on gastrointestinal motility and adrenocorticotropic hormone in normal controls and patients with irritable bowel syndrome. Gut 42, 845–849 10.1136/gut.42.6.845 9691924 PMC1727153

[B57] Gaman A. Kuo B. (2008). Neuromodulatory processes of the brain-gut axis. Neuromodulation 11, 249–259 10.1111/j.1525-1403.2008.00172.x 19844605 PMC2763396

[B58] Garvin B. Wiley J. W. (2008). The role of serotonin in irritable bowel syndrome: implications for management. Curr. Gastroenterol. Rep. 10, 363–368 10.1007/s11894-008-0070-3 18627647

[B59] Gershon M. D. Tack J. (2007). The serotonin signaling system: from basic understanding to drug development for functional GI disorders. Gastroenterology 132, 397–414 10.1053/j.gastro.2006.11.002 17241888

[B60] Greco B. Allegretto E. A. Tetel M. J. Blaustein J. D. (2001). Coexpression of ER beta with ER alpha and progestin receptor proteins in the female rat forebrain: effects of estradiol treatment. Endocrinology 142, 5172–5181 10.1210/en.142.12.5172 11713212

[B61] Greenwood-Van M. B. Gibson M. Gunter W. Shepard J. Foreman R. Myers D. (2001). Stereotaxic delivery of corticosterone to the amygdala modulates colonic sensitivity in rats. Brain Res. 893, 135–142 10.1016/S0006-8993(00)03305-9 11223001

[B62] Gros D. F. Antony M. M. McCabe R. E. Swinson R. P. (2009). Frequency and severity of the symptoms of irritable bowel syndrome across the anxiety disorders and depression. J. Anxiety Disord. 23, 290–296 10.1016/j.janxdis.2008.08.004 18819774

[B63] Grover M. Drossman D. A. (2011). Centrally acting therapies for irritable bowel syndrome. Gastroenterol. Clin. North Am. 40, 183–206 10.1016/j.gtc.2010.12.003 21333907

[B64] Grundmann O. Yoon S. L. Moshiree B. (2010). Current developments for the diagnosis and treatment of irritable bowel syndrome. Curr. Pharm. Des. 16, 3638–3645 10.2174/138161210794079227 21128902

[B65] Gue M. Junien J. L. Bueno L. (1991). Conditioned emotional response in rats enhances colonic motility through the central release of corticotropin-releasing factor. Gastroenterology 100, 964–970 2001832 10.1016/0016-5085(91)90270-u

[B66] Gustafsson J. K. Greenwood-Van M. B. (2011). Amygdala activation by corticosterone alters visceral and somatic pain in cycling female rats. Am. J. Physiol. Gastrointest. Liver Physiol. 300, G1080–G1085 10.1152/ajpgi.00349.2010 21454447

[B67] Gwee K. A. Leong Y. L. Graham C. McKendrick M. W. Collins S. M. Walters S. J. Underwood J. E. Read N. W. (1999). The role of psychological and biological factors in postinfective gut dysfunction. Gut 44, 400–406 10.1136/gut.44.3.400 10026328 PMC1727402

[B68] Hall G. B. Kamath M. V. Collins S. Ganguli S. Spaziani R. Miranda K. L. Bayati A. Bienenstock J. (2010). Heightened central affective response to visceral sensations of pain and discomfort in IBS. Neurogastroenterol. Motil. 22, 276–e80 10.1111/j.1365-2982.2009.01436.x 20003075

[B69] Herman J. P. Figueiredo H. Mueller N. K. Ulrich-Lai Y. Ostrander M. M. Choi D. C. Cullinan W. E. (2003). Central mechanisms of stress integration: hierarchical circuitry controlling hypothalamo-pituitary-adrenocortical responsiveness. Front. Neuroendocrinol. 24:7 10.1016/j.yfrne.2003.07.001 14596810

[B70] Houghton L. A. Lea R. Jackson N. Whorwell P. J. (2002). The menstrual cycle affects rectal sensitivity in patients with irritable bowel syndrome but not healthy volunteers. Gut 50, 471–474 10.1136/gut.50.4.471 11889064 PMC1773170

[B71] Hsu S. Y. Hsueh A. J. (2001). Human stresscopin and stresscopin-related peptide are selective ligands for the type 2 corticotropin-releasing hormone receptor. Nat. Med. 7, 605–611 10.1038/87936 11329063

[B72] Ibeakanma C. Ochoa-Cortes F. Miranda-Morales M. McDonald T. Spreadbury I. Cenac N. Ibeakanma C. Ochoa-Cortes F. Miranda-Morales M. McDonald T. Spreadbury I. Cenac N. Cattaruzza F. Hurlbut D. Vanner S. Bunnett N. Vergnolle N. Vanner S. (2011). Brain-gut interactions increase peripheral nociceptive signaling in mice with postinfectious irritable bowel syndrome. Gastroenterology 141, 2098–2108 10.1053/j.gastro.2011.08.006 21856270

[B73] Izzo A. A. Sharkey K. A. (2010). Cannabinoids and the gut: new developments and emerging concepts. Pharmacol. Ther. 126, 21–38 10.1016/j.pharmthera.2009.12.005 20117132

[B74] Jarcho J. M. Chang L. Berman M. Suyenobu B. Naliboff B. D. Lieberman M. D. Ameen V. Z. Mandelkern M. A. Mayer E. A. (2008). Neural and psychological predictors of treatment response in irritable bowel syndrome patients with a 5-HT3 receptor antagonist: a pilot study. Aliment. Pharmacol. Ther. 28, 344–352 10.1111/j.1365-2036.2008.03721.x 19086332 PMC2656431

[B75] Jarrett M. E. Burr R. L. Cain K. C. Hertig V. Weisman P. Heitkemper M. M. (2003). Anxiety and depression are related to autonomic nervous system function in women with irritable bowel syndrome. Dig. Dis. Sci. 48, 386–394 10.1023/A:1021904216312 12643620

[B76] John C. D. Buckingham J. C. (2003). Cytokines: regulation of the hypothalamo-pituitary-adrenocortical axis. Curr. Opin. Pharmacol. 3, 78–84 10.1016/S1471-4892(02)00009-7 12550746

[B77] Johnson A. C. Myers B. Lazovic J. Towner R. Greenwood-Van M. B. (2010). Brain activation in response to visceral stimulation in rats with amygdala implants of corticosterone: an FMRI study. PLoS ONE 5, e8573 10.1371/journal.pone.0015109 20052291 PMC2797306

[B78] Kane S. V. Sable K. Hanauer S. B. (1998). The menstrual cycle and its effect on inflammatory bowel disease and irritable bowel syndrome: a prevalence study. Am. J. Gastroenterol. 93, 1867–1872 10.1111/j.1572-0241.1998.540_i.x 9772046

[B79] Kennedy P. J. Clarke G. Quigley E. M. Groeger J. A. Dinan T. G. Cryan J. F. (2012). Gut memories: towards a cognitive neurobiology of irritable bowel syndrome. Neurosci. Biobehav. Rev. 36, 310–340 10.1016/j.neubiorev.2011.07.001 21777613

[B80] Khasar S. G. Burkham J. Dina O. A. Brown A. S. Bogen O. Alessandri-Haber N. Green P. G. Reichling D. B. Levine J. D. (2008). Stress induces a switch of intracellular signaling in sensory neurons in a model of generalized pain. J. Neurosci. 28, 5721–5730 10.1523/JNEUROSCI.0256-08.2008 18509033 PMC2518401

[B81] Kilpatrick L. A. Ornitz E. Ibrahimovic H. Treanor M. Craske M. Nazarian M. Kilpatrick L. A. Ornitz E. Ibrahimovic H. Treanor M. Craske M. Nazarian M. Labus J. S. Mayer E. A. Naliboff B. D. (2010). Sex-related differences in prepulse inhibition of startle in irritable bowel syndrome (IBS). Biol. Psychol. 84, 272–278 10.1016/j.biopsycho.2010.02.012 20193731 PMC2875286

[B82] Kirby L. G. Rice K. C. Valentino R. J. (2000). Effects of corticotropin-releasing factor on neuronal activity in the serotonergic dorsal raphe nucleus. Neuropsychopharmacology 22, 148–162 10.1016/S0893-133X(99)00093-7 10649828

[B83] Kostich W. A. Chen A. Sperle K. Largent B. L. (1998). Molecular identification and analysis of a novel human corticotropin-releasing factor (CRF) receptor: the CRF2gamma receptor. Mol. Endocrinol. 12, 1077–1085 10.1210/me.12.8.1077 9717834

[B84] Kozlowski C. M. Green A. Grundy D. Boissonade F. M. Bountra C. (2000). The 5-HT(3) receptor antagonist alosetron inhibits the colorectal distention induced depressor response and spinal c-fos expression in the anaesthetised rat. Gut 46, 474–480 10.1136/gut.46.4.474 10716675 PMC1727898

[B85] Kwan C. L. Diamant N. E. Pope G. Mikula K. Mikulis D. J. Davis K. D. (2005). Abnormal forebrain activity in functional bowel disorder patients with chronic pain. Neurology 65, 1268–1277 10.1212/01.wnl.0000180971.95473.cc 16247056

[B86] Labus J. S. Naliboff B. N. Fallon J. Berman S. M. Suyenobu B. Bueller J. A. Mandelkern M. Mayer E. A. (2008). Sex differences in brain activity during aversive visceral stimulation and its expectation in patients with chronic abdominal pain: a network analysis. Neuroimage 41, 1032–1043 10.1016/j.neuroimage.2008.03.009 18450481 PMC3114874

[B87] Lackner J. M. Lou C. M. Mertz H. R. Wack D. S. Katz L. A. Krasner S. S. Firth R. Mahl T. C. Lockwood A. H. (2006). Cognitive therapy for irritable bowel syndrome is associated with reduced limbic activity, GI symptoms, and anxiety. Behav. Res. Ther. 44, 621–638 10.1016/j.brat.2005.05.002 16039604 PMC6743496

[B88] Larauche M. Mulak A. Tache Y. (2012). Stress and visceral pain: from animal models to clinical therapies. Exp. Neurol. 233, 49–67 10.1016/j.expneurol.2011.04.020 21575632 PMC3224675

[B89] Lawal A. Kern M. Sidhu H. Hofmann C. Shaker R. (2006). Novel evidence for hypersensitivity of visceral sensory neural circuitry in irritable bowel syndrome patients. Gastroenterology 130, 26–33 10.1053/j.gastro.2005.10.020 16401465

[B90] Lee S. Y. Kim J. H. Sung I. K. Park H. S. Jin C. J. Choe W. H. Kwon S. Y. Lee C. H. Choi K. W. (2007). Irritable bowel syndrome is more common in women regardless of the menstrual phase: a Rome II-based survey. J. Korean Med. Sci. 22, 851–854 10.3346/jkms.2007.22.5.912 17982234 PMC2693852

[B91] Lembo T. Plourde V. Shui Z. Fullerton S. Mertz H. Tache Y. Sytnik B. Munakata J. Mayer E. (1996). Effects of the corticotropin-releasing factor (CRF) on rectal afferent nerves in humans. Neurogastroenterol. Motil. 8, 9–18 10.1111/j.1365-2982.1996.tb00237.x 8697187

[B92] Lewis K. Li C. Perrin M. H. Blount A. Kunitake K. Donaldson C. Vaughan J. Reyes T. M. Gulyas J. Fischer W. Bilezikjian L. Rivier J. Sawchenko P. E. Vale W. W. (2001). Identification of urocortin III, an additional member of the corticotropin-releasing factor (CRF) family with high affinity for the CRF2 receptor. Proc. Natl. Acad. Sci. U.S.A. 98, 7570–7575 10.1073/pnas.081075898 11416224 PMC34709

[B93] Lindstrom E. Ravnefjord A. Brusberg M. Hjorth S. Larsson H. Martinez V. (2009). The selective 5-hydroxytryptamine 1A antagonist, AZD7371 [3(R)-(N,N-dicyclobutylamino)-8-fluoro-3,4-dihydro-2H-1-benzopyran-5-carboxamide (R,R)-tartrate monohydrate] (robalzotan tartrate monohydrate), inhibits visceral pain-related visceromotor, but not autonomic cardiovascular, responses to colorectal distension in rats. J. Pharmacol. Exp. Ther. 329, 1048–1055 10.1124/jpet.109.152330 19325032

[B94] Lowry C. A. Moore F. L. (2006). Regulation of behavioral responses by corticotropin-releasing factor. Gen. Comp. Endocrinol. 146, 19–27 10.1016/j.ygcen.2005.12.006 16426606

[B95] Lukkes J. L. Forster G. L. Renner K. J. Summers C. H. (2008). Corticotropin-releasing factor 1 and 2 receptors in the dorsal raphe differentially affect serotonin release in the nucleus accumbens. Eur. J. Pharmacol. 578, 185–193 10.1016/j.ejphar.2007.09.024 17945210 PMC2276644

[B96] Mach T. (2004). The brain-gut axis in irritable bowel syndrome – clinical aspects. Med. Sci. Monit. 10, RA125–RA131 15173682

[B97] Manini M. L. Camilleri M. Goldberg M. Sweetser S. McKinzie S. Burton D. Wong S. Kitt M. M. Li Y. P. Zinsmeister A. R. (2010). Effects of Velusetrag (TD-5108) on gastrointestinal transit and bowel function in health and pharmacokinetics in health and constipation. Neurogastroenterol. Motil. 22, 42–48 19691492 10.1111/j.1365-2982.2009.01378.x PMC2905526

[B98] Martinez V. Tache Y. (2006). CRF1 receptors as a therapeutic target for irritable bowel syndrome. Curr. Pharm. Des. 12, 4071–4088 10.2174/138161206778743637 17100612

[B99] Martinez V. Wang L. Rivier J. Grigoriadis D. Tache Y. (2004). Central CRF, urocortins and stress increase colonic transit via CRF1 receptors while activation of CRF2 receptors delays gastric transit in mice. J. Physiol. (Lond.) 556(Pt 1), 221–234 10.1113/jphysiol.2003.059659 14755002 PMC1664879

[B100] Mayer E. A. Collins S. M. (2002). Evolving pathophysiologic models of functional gastrointestinal disorders. Gastroenterology 122, 2032–2048 10.1053/gast.2002.33584 12055608

[B101] Mayer E. A. Naliboff B. Lee O. Munakata J. Chang L. (1999). Review article: gender-related differences in functional gastrointestinal disorders. Aliment. Pharmacol. Ther. 13(Suppl. 2), 65–69 10.1046/j.1365-2036.1999.00008.x 10429743

[B102] Mayer E. A. Naliboff B. D. Craig A. D. (2006). Neuroimaging of the brain-gut axis: from basic understanding to treatment of functional GI disorders. Gastroenterology 131, 1925–1942 10.1053/j.gastro.2006.10.026 17188960

[B103] McEwen B. S. (2007). Physiology and neurobiology of stress and adaptation: central role of the brain. Physiol. Rev. 87, 873–904 10.1152/physrev.00041.2006 17615391

[B104] McKernan D. P. Gaszner G. Quigley E. M. Cryan J. F. Dinan T. G. (2011). Altered peripheral toll-like receptor responses in the irritable bowel syndrome. Aliment. Pharmacol. Ther. 33, 1045–1052 10.1111/j.1365-2036.2011.04624.x 21453321

[B105] McKernan D. P. Nolan A. Brint E. K. O’Mahony S. M. Hyland N. P. Cryan J. F. Dinan T. G. (2009). Toll-like receptor mRNA expression is selectively increased in the colonic mucosa of two animal models relevant to irritable bowel syndrome. PLoS ONE 4, e8226 10.1371/journal.pone.0008226 20011045 PMC2785428

[B106] Mertz H. Morgan V. Tanner G. Pickens D. Price R. Shyr Y. Kessler R. (2000). Regional cerebral activation in irritable bowel syndrome and control subjects with painful and nonpainful rectal distention. Gastroenterology 118, 842–848 10.1016/S0016-5085(00)85511-0 10784583

[B107] Meuser T. Pietruck C. Gabriel A. Xie G. X. Lim K. J. Pierce P. P. (2002). 5-HT7 receptors are involved in mediating 5-HT-induced activation of rat primary afferent neurons. Life Sci. 71, 2279–2289 10.1016/S0024-3205(02)02011-8 12215375

[B108] Million M. Grigoriadis D. E. Sullivan S. Crowe P. D. McRoberts J. A. Zhou H. Saunders P. R. Maillot C. Mayer E. A. Taché Y. (2003). A novel water-soluble selective CRF1 receptor antagonist, NBI 35965, blunts stress-induced visceral hyperalgesia and colonic motor function in rats. Brain Res. 985, 32–42 10.1016/S0006-8993(03)03027-0 12957366

[B109] Milner T. A. Lubbers L. S. Alves S. E. McEwen B. S. (2008). Nuclear and extranuclear estrogen binding sites in the rat forebrain and autonomic medullary areas. Endocrinology 149, 3306–3312 10.1210/en.2008-0307 18356271 PMC2453087

[B110] Moreau J. L. Kilpatrick G. Jenck F. (1997). Urocortin, a novel neuropeptide with anxiogenic-like properties. Neuroreport 8, 1697–1701 10.1097/00001756-199705060-00027 9189917

[B111] Morin S. M. Ling N. Liu X. J. Kahl S. D. Gehlert D. R. (1999). Differential distribution of urocortin- and corticotropin-releasing factor-like immunoreactivities in the rat brain. Neuroscience 92, 281–291 10.1016/S0306-4522(98)00732-5 10392850

[B112] Mulak A. Bonaz B. (2004). Irritable bowel syndrome: a model of the brain-gut interactions. Med. Sci. Monit. 10, RA55–RA62 15260348

[B113] Mulak A. Paradowski L. (2006). Effect of 5-HT1 agonist (sumatriptan) on anorectal function in irritable bowel syndrome patients. World J. Gastroenterol. 12, 1591–1596 16570352 10.3748/wjg.v12.i10.1591 PMC4124292

[B114] Myers B. Greenwood-Van M. B. (2007). Corticosteroid receptor-mediated mechanisms in the amygdala regulate anxiety and colonic sensitivity. Am. J. Physiol. Gastrointest. Liver Physiol. 292, G1622–G1629 10.1152/ajpgi.00080.2007 17347454

[B115] Myers B. Greenwood-Van M. B. (2009). Role of anxiety in the pathophysiology of irritable bowel syndrome: importance of the amygdala. Front. Neurosci. 3:47 10.3389/neuro.21.002.2009 20582274 PMC3112316

[B116] Myers B. Greenwood-Van M. B. (2010). Elevated corticosterone in the amygdala leads to persistent increases in anxiety-like behavior and pain sensitivity. Behav. Brain Res. 214, 465–469 10.1016/j.bbr.2010.05.049 20573588

[B117] Myers B. Schulkin J. Greenwood-Van M. B. (2011). Sex steroids localized to the amygdala increase pain responses to visceral stimulation in rats. J. Pain. 12, 486–494 10.1016/j.jpain.2010.10.007 21167789

[B118] Naliboff B. D. Berman S. Chang L. Derbyshire S. W. Suyenobu B. Vogt B. A. Mandelkern M. Mayer E. A. (2003). Sex-related differences in IBS patients: central processing of visceral stimuli. Gastroenterology 124, 1738–1747 10.1016/S0016-5085(03)00400-1 12806606

[B119] Naliboff B. D. Derbyshire S. W. Munakata J. Berman S. Mandelkern M. Chang L. Mayer E. A. (2001). Cerebral activation in patients with irritable bowel syndrome and control subjects during rectosigmoid stimulation. Psychosom. Med. 63, 365–375 11382264 10.1097/00006842-200105000-00006

[B120] Niddam D. M. Tsai S. Y. Lu C. L. Ko C. W. Hsieh J. C. (2011). Reduced hippocampal glutamate-glutamine levels in irritable bowel syndrome: preliminary findings using magnetic resonance spectroscopy. Am. J. Gastroenterol. 106, 1503–1511 10.1038/ajg.2011.120 21502999

[B121] Ohman L. Simren M. (2010). Pathogenesis of IBS: role of inflammation, immunity and neuroimmune interactions. Nat. Rev. Gastroenterol. Hepatol. 7, 163–173 10.1038/nrgastro.2010.4 20101257

[B122] O’Mahony S. M. Bulmer D. C. Coelho A. M. Fitzgerald P. Bongiovanni C. Lee K. Winchester W. Dinan T. G. Cryan J. F. (2010a). 5-HT(2B) receptors modulate visceral hypersensitivity in a stress-sensitive animal model of brain-gut axis dysfunction. Neurogastroenterol. Motil. 22, 573–578, e124. 10.1111/j.1365-2982.2009.01432.x 20003079

[B123] O’Mahony S. M. Bulmer D. C. Coelho A. M. Fitzgerald P. Bongiovanni C. Lee K. Winchester W. Dinan T. G. Cryan J. F. (2010b). 5-HT(2B) receptors modulate visceral hypersensitivity in a stress-sensitive animal model of brain-gut axis dysfunction. Neurogastroenterol. Motil. 22, 573–578, e124. 10.1111/j.1365-2982.2009.01432.x 20003079

[B124] O’Mahony S. M. Hyland N. P. Dinan T. G. Cryan J. F. (2011). Maternal separation as a model of brain-gut axis dysfunction. Psychopharmacology (Berl.) 214, 71–88 10.1007/s00213-010-2010-9 20886335

[B125] Owens M. J. Nemeroff C. B. (1993). The role of corticotropin-releasing factor in the pathophysiology of affective and anxiety disorders: laboratory and clinical studies. Ciba Found. Symp. 172, 296–308 8491091 10.1002/9780470514368.ch15

[B126] Pandey S. C. Zhang H. Roy A. Misra K. (2006). Central and medial amygdaloid brain-derived neurotrophic factor signaling plays a critical role in alcohol-drinking and anxiety-like behaviors. J. Neurosci. 26, 8320–8331 10.1523/JNEUROSCI.4988-05.2006 16899727 PMC6673799

[B127] Penalva R. G. Flachskamm C. Zimmermann S. Wurst W. Holsboer F. Reul J. M. Linthorst A. C. (2002). Corticotropin-releasing hormone receptor type 1-deficiency enhances hippocampal serotonergic neurotransmission: an in vivo microdialysis study in mutant mice. Neuroscience 109, 253–266 10.1016/S0306-4522(01)00475-4 11801362

[B128] Philpott H. Gibson P. Thien F. (2011). Irritable bowel syndrome – an inflammatory disease involving mast cells. Asia Pac. Allergy 1, 36–42 10.5415/apallergy.2011.1.1.36 22053295 PMC3206231

[B129] Piche M. Bouin M. Arsenault M. Poitras P. Rainville P. (2011). Decreased pain inhibition in irritable bowel syndrome depends on altered descending modulation and higher-order brain processes. Neuroscience 195, 166–175 10.1016/j.neuroscience.2011.08.040 21889972

[B130] Prado W. A. Roberts M. H. (1985). An assessment of the antinociceptive and aversive effects of stimulating identified sites in the rat brain. Brain Res. 340, 219–228 10.1016/0006-8993(85)90917-5 4027651

[B131] Price M. L. Curtis A. L. Kirby L. G. Valentino R. J. Lucki I. (1998). Effects of corticotropin-releasing factor on brain serotonergic activity. Neuropsychopharmacology 18, 492–502 10.1016/S0893-133X(97)00197-8 9571657

[B132] Rapps N. van Oudenhove L. Enck P. Aziz Q. (2008). Brain imaging of visceral functions in healthy volunteers and IBS patients. J. Psychosom. Res. 64, 599–604 10.1016/j.jpsychores.2008.02.018 18501260

[B133] Redgate E. S. Fahringer E. E. (1973). A comparison of the pituitary adrenal activity elicited by electrical stimulation of preoptic, amygdaloid and hypothalamic sites in the rat brain. Neuroendocrinology 12, 334–343 10.1159/000122182 4760260

[B134] Ren T. H. Wu J. Yew D. Ziea E. Lao L. Leung W. K. Berman B. Hu P. J. Sung J. J. (2007). Effects of neonatal maternal separation on neurochemical and sensory response to colonic distension in a rat model of irritable bowel syndrome. Am. J. Physiol. Gastrointest. Liver Physiol. 292, G849–G856 10.1152/ajpgi.00400.2006 17110521

[B135] Reyes T. M. Lewis K. Perrin M. H. Kunitake K. S. Vaughan J. Arias C. A. Hogenesch J. B. Gulyas J. Rivier J. Vale W. W. Sawchenko P. E. (2001). Urocortin II: a member of the corticotropin-releasing factor (CRF) neuropeptide family that is selectively bound by type 2 CRF receptors. Proc. Natl. Acad. Sci. U.S.A. 98, 2843–2848 10.1073/pnas.051626398 11226328 PMC30227

[B136] Ribeiro S. C. Kennedy S. E. Smith Y. R. Stohler C. S. Zubieta J. K. (2005). Interface of physical and emotional stress regulation through the endogenous opioid system and mu-opioid receptors. Prog. Neuropsychopharmacol. Biol. Psychiatry 29, 1264–1280 10.1016/j.pnpbp.2005.08.011 16256255

[B137] Ringel Y. (2002). Brain research in functional gastrointestinal disorders. J. Clin. Gastroenterol. 35(1 Suppl.), S23–S25 10.1097/00004836-200207001-00003 12184135

[B138] Ringel Y. Drossman D. A. Leserman J. L. Suyenobu B. Y. Wilber K. Lin W. Whitehead W. E. Naliboff B. D. Berman S. Mayer E. A. (2008). Effect of abuse history on pain reports and brain responses to aversive visceral stimulation: an FMRI study. Gastroenterology 134, 396–404 10.1053/j.gastro.2007.11.011 18242208

[B139] Rosas-Ballina M. Tracey K. J. (2009). The neurology of the immune system: neural reflexes regulate immunity. Neuron 64, 28–32 10.1016/j.neuron.2009.09.039 19840545 PMC4533851

[B140] Sagami Y. Shimada Y. Tayama J. Nomura T. Satake M. Endo Y. Shoji T. Karahashi K. Hongo M. Fukudo S. (2004). Effect of a corticotropin releasing hormone receptor antagonist on colonic sensory and motor function in patients with irritable bowel syndrome. Gut 53, 958–964 10.1136/gut.2003.018911 15194643 PMC1774093

[B141] Saito K. Kanazawa M. Fukudo S. (2002). Colorectal distention induces hippocampal noradrenaline release in rats: an in vivo microdialysis study. Brain Res. 947, 146–149 10.1016/S0006-8993(02)03007-X 12144863

[B142] Salari P. Abdollahi M. (2011). Systematic review of modulators of benzodiazepine receptors in irritable bowel syndrome: is there hope? World J. Gastroenterol. 17, 4251–4257 10.3748/wjg.v17.i38.4251 22090780 PMC3214699

[B143] Sandler R. S. Everhart J. E. Donowitz M. Adams E. Cronin K. Goodman C. Gemmen E. Shah S. Avdic A. Rubin R. (2002). The burden of selected digestive diseases in the United States. Gastroenterology 122, 1500–1511 10.1053/gast.2002.32978 11984534

[B144] Saunders P. R. Maillot C. Million M. Tache Y. (2002). Peripheral corticotropin-releasing factor induces diarrhea in rats: role of CRF1 receptor in fecal watery excretion. Eur. J. Pharmacol. 435, 231–235 10.1016/S0014-2999(01)01574-6 11821031

[B145] Seminowicz D. A. Labus J. S. Bueller J. A. Tillisch K. Naliboff B. D. Bushnell M. C. Mayer E. A. (2010). Regional gray matter density changes in brains of patients with irritable bowel syndrome. Gastroenterology 139, 48–57 10.1053/j.gastro.2010.03.049 20347816 PMC2902717

[B146] Shepard J. D. Barron K. W. Myers D. A. (2003). Stereotaxic localization of corticosterone to the amygdala enhances hypothalamo-pituitary-adrenal responses to behavioral stress. Brain Res. 963, 203–213 10.1016/S0006-8993(02)03978-1 12560126

[B147] Sivarao D. V. Newberry K. Lodge N. J. (2004). Effect of the 5HT1A receptor partial agonist buspirone on colorectal distension-induced pseudoaffective and behavioral responses in the female Wistar rat. Eur. J. Pharmacol. 494, 23–29 10.1016/j.ejphar.2004.04.034 15194447

[B148] Slack S. E. Pezet S. McMahon S. B. Thompson S. W. Malcangio M. (2004). Brain-derived neurotrophic factor induces NMDA receptor subunit one phosphorylation via ERK and PKC in the rat spinal cord. Eur. J. Neurosci. 20, 1769–1778 10.1111/j.1460-9568.2004.03656.x 15379998

[B149] Slawecki C. J. Somes C. Rivier J. E. Ehlers C. L. (1999). Neurophysiological effects of intracerebroventricular administration of urocortin. Peptides 20, 211–218 10.1016/S0196-9781(98)00160-0 10422877

[B150] Smith G. W. Aubry J. M. Dellu F. Contarino A. Bilezikjian L. M. Gold L. H. Chen R. Marchuk Y. Hauser C. Bentley C. A. Sawchenko P. E. Koob G. F. Vale W. Lee K. F. (1998). Corticotropin releasing factor receptor 1-deficient mice display decreased anxiety, impaired stress response, and aberrant neuroendocrine development. Neuron 20, 1093–1102 10.1016/S0896-6273(00)80504-8 9655498

[B151] Southwick S. M. Bremner J. D. Rasmusson A. Morgan C. A. III Arnsten A. Charney D. S. (1999). Role of norepinephrine in the pathophysiology and treatment of posttraumatic stress disorder. Biol. Psychiatry 46, 1192–1204 10.1016/S0006-3223(99)00107-9 10560025

[B152] Spaziani R. Bayati A. Redmond K. Bajaj H. Mazzadi S. Bienenstock J. Collins S. M. Kamath M. V. (2008). Vagal dysfunction in irritable bowel syndrome assessed by rectal distension and baroreceptor sensitivity. Neurogastroenterol. Motil. 20, 336–342 10.1111/j.1365-2982.2007.01042.x 18179607

[B153] Spiller R. (2007). Recent advances in understanding the role of serotonin in gastrointestinal motility in functional bowel disorders: alterations in 5-HT signalling and metabolism in human disease. Neurogastroenterol. Motil. 19(Suppl. 2), 25–31 10.1111/j.1365-2982.2007.00965.x 17620085

[B154] Spiller R. Garsed K. (2009). Infection, inflammation, and the irritable bowel syndrome. Dig. Liver Dis. 41, 844–849 10.1016/j.dld.2009.07.007 19716778

[B155] Sweetser S. Camilleri M. Linker Nord S. J. Burton D. D. Castenada L. Croop R. Tong G. Dockens R. Zinsmeister A. R. (2009). Do corticotropin releasing factor-1 receptors influence colonic transit and bowel function in women with irritable bowel syndrome? Am. J. Physiol. Gastrointest. Liver Physiol. 296, G1299–G1306 10.1152/ajpgi.00011.2009 19342506 PMC2697942

[B156] Tache Y. Brunnhuber S. (2008). From Hans Selye’s discovery of biological stress to the identification of corticotropin-releasing factor signaling pathways: implication in stress-related functional bowel diseases. Ann. N. Y. Acad. Sci. 1148, 29–41 10.1196/annals.1410.007 19120089 PMC2993154

[B157] Tache Y. Kiank C. Stengel A. (2009). A role for corticotropin-releasing factor in functional gastrointestinal disorders. Curr. Gastroenterol. Rep. 11, 270–277 10.1007/s11894-009-0040-4 19615302 PMC3295847

[B158] Tache Y. Martinez V. Million M. Maillot C. (2002). Role of corticotropin releasing factor receptor subtype 1 in stress-related functional colonic alterations: implications in irritable bowel syndrome. Eur. J. Surg. 168(Suppl. 1), 16–22 16144197

[B159] Tache Y. Martinez V. Wang L. Million M. (2004). CRF1 receptor signaling pathways are involved in stress-related alterations of colonic function and viscerosensitivity: implications for irritable bowel syndrome. Br. J. Pharmacol. 141, 1321–1330 10.1038/sj.bjp.0705760 15100165 PMC1574904

[B160] Tack J. Broekaert D. Fischler B. van Oudenhove L. Gevers A. M. Janssens J. (2006). A controlled crossover study of the selective serotonin reuptake inhibitor citalopram in irritable bowel syndrome. Gut 55, 1095–1103 10.1136/gut.2004.060426 16401691 PMC1856276

[B161] Tack J. Coulie B. Wilmer A. Andrioli A. Janssens J. (2000). Influence of sumatriptan on gastric fundus tone and on the perception of gastric distension in man. Gut 46, 468–473 10.1136/gut.46.4.468 10716674 PMC1727890

[B162] Takeuchi O. Akira S. (2010). Pattern recognition receptors and inflammation. Cell 140, 805–820 10.1016/j.cell.2010.01.022 20303872

[B163] Thompson W. G. Heaton K. W. Smyth G. T. Smyth C. (2000). Irritable bowel syndrome in general practice: prevalence, characteristics, and referral. Gut 46, 78–82 10.1136/gut.46.1.78 10601059 PMC1727778

[B164] Tillisch K. Labus J. Nam B. Bueller J. Smith S. Suyenobu B. Siffert J. McKelvy J. Naliboff B. Mayer E. (2012). Neurokinin-1-receptor antagonism decreases anxiety and emotional arousal circuit response to noxious visceral distension in women with irritable bowel syndrome: a pilot study. Aliment. Pharmacol. Ther. 35, 360–367 10.1111/j.1365-2036.2011.04958.x 22221140 PMC4073664

[B165] Tillisch K. Mayer E. A. Labus J. S. (2011). Quantitative meta-analysis identifies brain regions activated during rectal distension in irritable bowel syndrome. Gastroenterology 140, 91–100 10.1053/j.gastro.2010.07.053 20696168 PMC3253553

[B166] Tillisch K. Mayer E. A. Labus J. S. Stains J. Chang L. Naliboff B. D. (2005). Sex specific alterations in autonomic function among patients with irritable bowel syndrome. Gut 54, 1396–1401 10.1136/gut.2004.058685 15923667 PMC1774694

[B167] Tjong Y. W. Ip S. P. Lao L. Wu J. Fong H. H. Sung J. J. Berman B. Che C. T. (2010). Neonatal maternal separation elevates thalamic corticotrophin releasing factor type 1 receptor expression response to colonic distension in rat. Neuro Endocrinol. Lett. 31, 215–220 20424584

[B168] Toner B. B. Segal Z. V. Emmott S. Myran D. Ali A. DiGasbarro I. Stuckless N. (1998). Cognitive-behavioral group therapy for patients with irritable bowel syndrome. Int. J. Group Psychother. 48, 215–243 9563239 10.1080/00207284.1998.11491537

[B169] Trimble N. Johnson A. C. Foster A. Greenwood-Van M. B. (2007). Corticotropin-releasing factor receptor 1-deficient mice show decreased anxiety and colonic sensitivity. Neurogastroenterol. Motil. 19, 754–760 10.1111/j.1365-2982.2007.00951.x 17539891

[B170] Tyler K. Moriceau S. Sullivan R. M. Greenwood-Van M. B. (2007). Long-term colonic hypersensitivity in adult rats induced by neonatal unpredictable vs predictable shock. Neurogastroenterol. Motil. 19, 761–768 10.1111/j.1365-2982.2007.00955.x 17727395 PMC1964755

[B171] Vale W. Spiess J. Rivier C. Rivier J. (1981). Characterization of a 41-residue ovine hypothalamic peptide that stimulates secretion of corticotropin and beta-endorphin. Science 213, 1394–1397 10.1126/science.6267699 6267699

[B172] Valentino R. J. Commons K. G. (2005). Peptides that fine-tune the serotonin system. Neuropeptides 39, 1–8 10.1016/j.npep.2004.09.005 15627494

[B173] Vanhoenacker P. Haegeman G. Leysen J. E. (2000). 5-HT7 receptors: current knowledge and future prospects. Trends Pharmacol. Sci. 21, 70–77 10.1016/S0165-6147(99)01432-7 10664612

[B174] Venkova K. Johnson A. C. Myers B. Greenwood-Van M. B. (2010). Exposure of the amygdala to elevated levels of corticosterone alters colonic motility in response to acute psychological stress. Neuropharmacology 58, 1161–1167 10.1016/j.neuropharm.2010.02.012 20170666

[B175] Vera-Portocarrero L. P. Ossipov M. H. King T. Porreca F. (2008). Reversal of inflammatory and noninflammatory visceral pain by central or peripheral actions of sumatriptan. Gastroenterology 135, 1369–1378 10.1053/j.gastro.2008.06.085 18694754 PMC4028637

[B176] Vetter D. E. Li C. Zhao L. Contarino A. Liberman M. C. Smith G. W. Marchuk Y. Koob G. F. Heinemann S. F. Vale W. Lee K. F. (2002). Urocortin-deficient mice show hearing impairment and increased anxiety-like behavior. Nat. Genet. 31, 363–369 12091910 10.1038/ng914

[B177] Videlock E. J. Adeyemo M. Licudine A. Hirano M. Ohning G. Mayer M. Mayer E. A. Chang L. (2009). Childhood trauma is associated with hypothalamic-pituitary-adrenal axis responsiveness in irritable bowel syndrome. Gastroenterology 137, 1954–1962 10.1053/j.gastro.2009.08.058 19737564 PMC2789911

[B178] Wilder-Smith C. H. Schindler D. Lovblad K. Redmond S. M. Nirkko A. (2004). Brain functional magnetic resonance imaging of rectal pain and activation of endogenous inhibitory mechanisms in irritable bowel syndrome patient subgroups and healthy controls. Gut 53, 1595–1601 10.1136/gut.2003.028514 15479679 PMC1774298

[B179] Winston J. H. Xu G. Y. Sarna S. K. (2010). Adrenergic stimulation mediates visceral hypersensitivity to colorectal distension following heterotypic chronic stress. Gastroenterology 138, 294–304 10.1016/S0016-5085(10)63555-X 19800336 PMC2813397

[B180] Yu Y. B. Zuo X. L. Zhao Q. J. Chen F. X. Yang J. Dong Y. Y. Wang P. Li Y.-Q. (2012). Brain-derived neurotrophic factor contributes to abdominal pain in irritable bowel syndrome. Gut 61, 685–694 10.1136/gutjnl-2011-300265 21997550

[B181] Zisterer D. M. Williams D. C. (1997). Peripheral-type benzodiazepine receptors. Gen. Pharmacol. 29, 305–314 10.1016/S0306-3623(96)00473-9 9378234

[B182] Zou B. C. Dong L. Wang Y. Wang S. H. Cao M. B. (2007). Expression and role of 5-HT7 receptor in brain and intestine in rats with irritable bowel syndrome. Chin. Med. J. 120, 2069–2074 18167178

